# Clinical and Cosmetic Results After Double-Row Reconstruction of the Distal Triceps Tendon in an Athletic Population: A Retrospective Case Series of 70 Patients With a Mean Follow-up of 6 Years

**DOI:** 10.1177/03635465251389010

**Published:** 2026-01-01

**Authors:** Lorenz Fritsch, Lucca Lacheta, Nicolas Kühne, Sebastian Lappen, Maximilian Hinz, Sebastian Siebenlist, Mathias Ritsch

**Affiliations:** * Department of Sports Orthopaedics, Technical University of Munich, Munich, Germany; † The Steadman Philippon Research Institute, Vail, Colorado, USA; § Department for Shoulder and Elbow Surgery, Schulthess Clinic, Zürich, Switzerland; ‖ Sportortho-ro, Schön-Klinik Vogtareuth, Rosenheim, Germany; Investigation performed at Department of Sports Orthopaedics, Technical University of Munich and Sportortho-ro, Schön-Klinik Vogtareuth, Germany

**Keywords:** clinical medicine by anatomic region, clinical medicine by specialty interest, elbow, general sports trauma, injuries/conditions related to specific sports, weight lifting

## Abstract

**Background:**

Clinical outcomes after surgical repair of the distal triceps tendon are scarce and represented in small, heterogeneous case series.

**Purpose:**

To evaluate clinical and cosmetic outcomes after double-row repair in a high-demand athlete population.

**Study Design:**

Case series; Level of evidence, 4.

**Methods:**

All patients who participated in regular weight lifting and underwent distal triceps tendon repairs between 2000 and 2021 in 2 centers were retrospectively contacted for informed consent and follow-up examination. Patients who received distal triceps tendon repair in double-row fashion with a minimum follow-up of 24 months were included. The American Shoulder and Elbow Surgeons (ASES) score, Single Assessment Numeric Evaluation (SANE) score, Mayo Elbow Performance Score (MEPS; without instability), and Disabilities of the Arm, Shoulder and Hand (DASH) score were surveyed. General satisfaction on a scale from 0 (very unsatisfied) to 10 (very satisfied) was evaluated. In addition, a customized sporting activities questionnaire including subjective strength perception (0%-100%), time to return to sport, sports performance (bench and triceps press), visual analog scale (VAS) pain score, cosmetic results, complications, and failures (rerupture or reoperation) was administered.

**Results:**

A total of 70 patients (all male) with a mean age of 50.9 ± 8.7 years were included in this study. The mean follow-up was 86.9 ± 51.4 months. The postoperative outcome scores were as follows: 97.8 ± 4.8 for the ASES score, 93.6 ± 10.9 for the SANE score, 2.2 ± 5.5 for the DASH score, and 98.1 ± 6.4 points for the MEPS. The median satisfaction score was 10 (IQR, 10-10). Postoperatively, patients subjectively achieved a 94% return of prior strength after a median of 7 months. In bench and triceps press, pre- to postoperative weight loads were a mean of 162.03 ± 53.1 kg to 134.7 ± 52.1 kg (*P* = .001) and a median of 70 kg (IQR, 50-85 kg) to 60 kg (IQR, 50-60 kg) (*P* = .001), respectively. The preoperative VAS score was 5.7 ± 2.7 versus 0.2 ± 0.6 postoperatively (*P* = .001). Overall, 85.7% of patients were satisfied with the cosmetic result. In total, 6 reruptures (8.6%) and 1 infection (1.4%) were observed. All 7 patients underwent surgical revision.

**Conclusion:**

Double-row reconstruction of distal triceps tendon ruptures achieved good clinical and cosmetic results with a low complication rate in this high-demand patient population. Subjectively, maximum strength was regained after a median of 7 months; however, selective triceps strength during bench and triceps press resulted in significantly reduced weight loads postoperatively.

A rupture of the triceps tendon is among the rarest muscle-tendon injuries, comprising only 2% of all upper limb tendon injuries and only 0.8% of all tendon injuries,^3,5,11,14^ affecting mostly male patients between 30 and 50 years of age.^8,21^ Falling on an outstretched hand or sporting activities like weight lifting or body building represent the most frequent injury patterns.^12,21,29^ Systematic rheumatic or kidney diseases as well as the intake of anabolic steroids increase the risk of rupture.^12,21,22,27-29^

In the management of triceps tendon injuries, nonoperative treatment may be a viable option for partial ruptures.^13^ However, patient age, comorbidities, and activity level have to be considered.^18^

Complete ruptures or high-grade partial tears involving >50% of the tendon generally require surgical treatment with transosseous tunnels or suture anchors.^3,17^ Looking at both treatment options, early surgical treatment shows superior results compared with nonoperative treatment for both full-thickness and partial ruptures of the tendon.^23^

Several techniques have been described in the literature to surgically address distal triceps tendon rupture. Besides transosseous repair techniques, suture anchor reconstruction in a double-row fashion has recently gained attention in the literature.^4,7^ Biomechanical studies have demonstrated a higher load to failure when refixing the distal triceps tendon with a double-row suture anchor technique.^9,10,26^

Reported patient numbers after distal triceps repair are limited^19,29^ with heterogeneous surgical techniques.^16,30^ There is a lack of reported return-to-sports rate, especially in terms of triceps-demanding sports such as weight lifting.^1,2^

Therefore, the purpose of this study was to evaluate clinical and cosmetic outcomes after distal triceps tendon repair in a double-row manner and analyze the patients’ return to sports and return to competition, as well as specifically examine their postoperative ability to perform triceps-demanding sports exercises.

We hypothesized that double-row reconstruction of the distal triceps tendon would lead to beneficial clinical results and enable patients to return to sports at a high level.

## Methods

This retrospective bicentric outcome study was approved by our institutional review boards. Two institutions obtained a data bank of surgeries performed between 2000 and 2021. Those were filtered for patients who matched the following inclusion criteria: aged 18 years or older at the time of surgery, experienced an acute or chronic (lesions treated unsuccessfully by physical therapy) distal triceps tear and underwent open repair of the triceps tendon with a double-row reconstruction technique with a minimum follow-up of 24 months, and actively took part in strength training with active (or formerly active) participation in competitions (eg, body building competitions or weight lifting competition). Every patient was diligently informed and had to provide informed consent.

### Surgical Technique

Surgeries were performed as double-row triceps tendon reconstruction, as previously described.^24^

**Figure 1.**
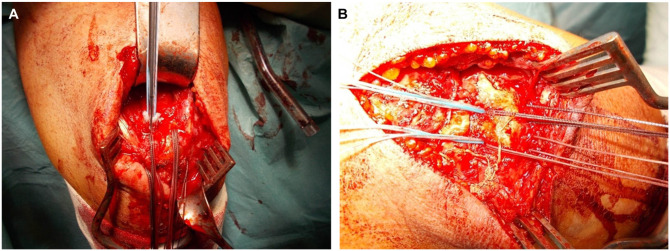
(A) Intraoperative view of the placement of the proximal anchors. (B) Final construct with proximal sutures and distal transosseous sutures.

**Figure 2.**
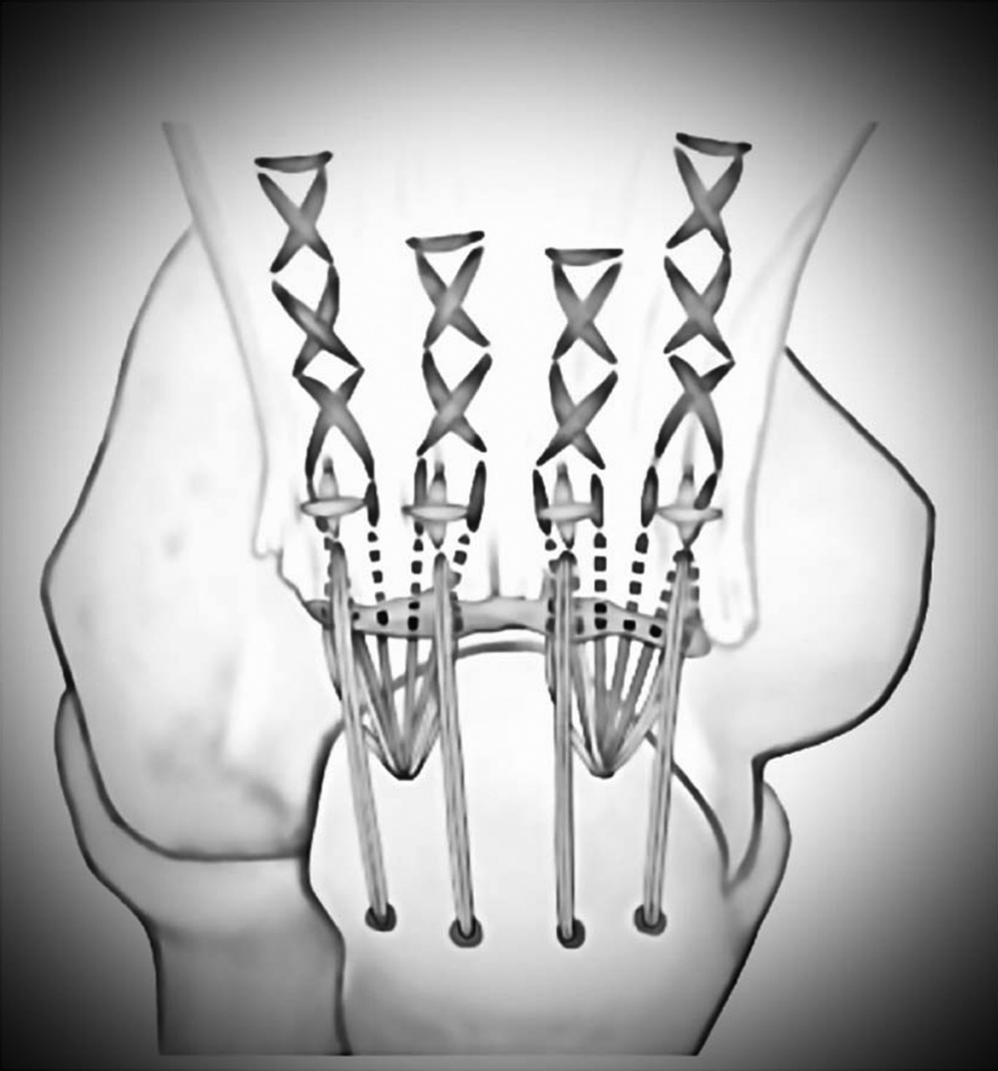
Double-row reconstruction illustrating the proximal suture anchor row and the additional transosseous repair technique. Modified after Ritsch et al.^24^ with permission (License number 6125620703312).

The patient was placed in a prone or side position and received general anesthesia. A standard posterior approach was used to visualize the ruptured tendon and the footprint, directed laterally around the olecranon. The tendon was mobilized and the olecranon was debrided. Then, two 2.9-mm holes were drilled, medially and laterally, into the proximal olecranon footprint to accommodate suture anchors. Additionally, a transosseous drilling tunnel was placed through the olecranon about 20 mm distal to the tip, and 4 sutures were passed through this tunnel (Figure 1). Next, suture anchors were inserted into the drilled holes (either all-suture or titanium) (Figure 2). The suture anchors enabled the surgeon to address both the superficial and the deep layer of the triceps tendon. The more proximal tendon portions were refixed with transosseous sutures.

### Postoperative Rehabilitation

Immediately postoperatively, an anterior splint at 10° of extension was applied. After that, an orthosis fixed in 20° of extension, allowing passive motion between 0° and 30° of flexion when putting the splint away for exercise reasons, was applied for 6 weeks. Starting in week 7, load-free, physical therapist–guided mobilization was allowed, gradually increasing the range of motion. Weightbearing began in week 13 postoperatively. Full loading was permitted after 6 months.

### Clinical Evaluation

Patient-reported outcomes were evaluated using the American Shoulder and Elbow Surgeons (ASES) score, Single Assessment Numeric Evaluation (SANE) score, Disabilities of the Arm, Shoulder and Hand questionnaire (DASH) score, Mayo Elbow Performance Score (MEPS), and visual analog scale (VAS) pain score. For the MEPS, the subscale stability was excluded as patients were only contacted via mail. General satisfaction on a scale of 0 (not satisfied) to 10 (very satisfied) was evaluated. In order to assess the range of motion of each patient, exemplary pictures were provided in the questionnaire where patients could choose the images referring to their actual range of motion.

### Return to Sports and Cosmesis Assessment

A custom questionnaire was designed to evaluate patients’ return to sports and return to competition. Patients were asked to complete questions concerning their level of sports before surgery and at time of follow-up. Therefore, they had to specify their prior and recent sporting activity, including hours of practice per week. Furthermore, they were questioned if they participated in competition before and after surgery. In addition, the questionnaire included pre- and postoperative performance questions focusing on exercises particularly involving the triceps. Patients were obliged to state their levels in classic bench press and triceps press. Lastly, patients rated their subjective cosmetic results in terms of persistent muscle deformity (the “reverse pop-eye” sign) or scar deformity, indicating whether they were satisfied or dissatisfied.

### Complications

By screening the postoperative documentation and part of the customized questionnaire, the occurrences of peri- and postoperative complications and failures were assessed. Failure was defined as structural rerupture and/or reoperation of the distal triceps tendon.

### Statistical Analysis

Descriptive statistics are used to summarize categorical and continuous variables, with categorical variables reported as counts and percentages and continuous variables reported as mean ± standard deviation or median and interquartile range. Continuous variables were analyzed using a Kolmogorov-Smirnoff test to evaluate their normal distribution. When comparing variables, a paired *t* test or Mann-Whitney *U* test was utilized. The significance level was set at a *P* value <.05. Statistical analysis was performed using IBM SPSS software (version 30.0; IBM).

## Results

**Figure 3.**
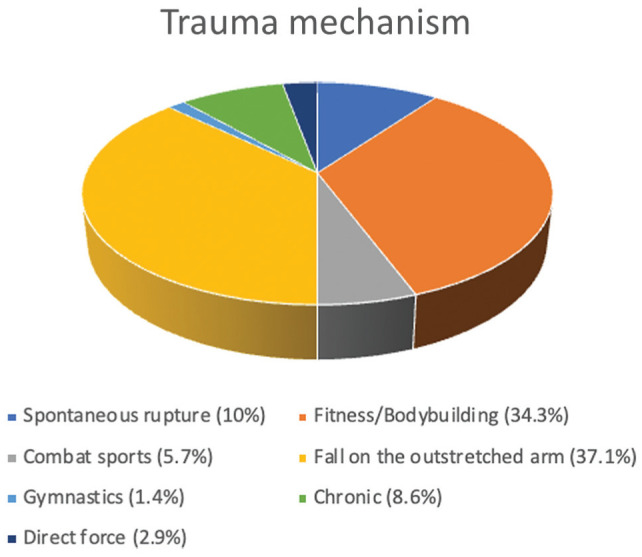
Trauma mechanisms for triceps rupture.

A total of 70 patients were included in the final analysis. The mean follow-up was 86.9 ± 51.4 months (range, 37-143 months). All patients were male. The mean age was 50.9 ± 8.7 years. Overall, 44% of triceps ruptures affected the right side, 56% left side, and in 54.3% of cases the dominant elbow was affected. A total of 22 patients (31.4%) had consecutive distal triceps repair of both arms. In 37.1% of cases, falling on the outstretched arm was the determining factor for the triceps tendon rupture. Further trauma mechanisms included strength sports (34.3%), spontaneous ruptures (10%), chronic ruptures (8.6%), combat sports (5.7%), direct force to the elbow (2.9%) and gymnastics (1.4%). (Figure 3).

Patient and surgical characteristics of the population are provided in Table 1.

**Table 1:** Patient and Surgical Characteristics*^*a*^*

	Value
Sex	
Male	70
Female	0
Age at time of surgery, y	50.9 ± 8.7
Follow-up, mo	86.9 ± 51.4
Affected side	
Right side	31 (44.3)
Left side	39 (55.7)
Dominant elbow	38 (54.3)
Preexisting pain at the operated triceps	30 (42.9)
Rupture of contralateral triceps	22 (31.4)
Rupture morphology	
Full-thickness tear	15 (21.4)
Partial rupture	55 (78.6)
Rupture of caput longum and laterale	47 (85.5)
Rupture of isolated caput longum	8 (14.5)

a Data are presented as n (%) or mean ± SD.

### Clinical Outcome

The mean postoperative ASES score was 97.8 ± 4.8, while the mean SANE score was 93.6 ± 10.9. In the final assessment, the mean DASH score was 2.2 ± 5.5, and patients achieved a mean MEPS of 98.1 ± 6.4. Pain levels, as measured by the VAS, showed a significant reduction from the pre- to postoperative evaluation (5.7 ± 2.7 to 0.2 ± 0.6; *P* = .001). The median patient satisfaction score was 10 (IQR, 10–10). Also, no patient reported postoperative subjective extension deficit.

### Return to Sports and Cosmesis

A total of 66 patients (94.3%) were able to return to sports; 64 patients (91.4%) could return to their prior sports activity. Of the 70 patients, 64 (91.4%) named body building or other strength sports as their main activity, 4 patients (5.7%) participated in combat sports, and 2 patients (2.9%) participated in other sports. Of all patients, 36 patients (51.4%) participated actively in body building at a competitive level before surgery. Of those, 20 patients (55.6%) were able to rejoin competitive sports, with 90% at their prior competitive level. At the time of follow-up, patients subjectively rated their extension strength at a mean of 93.6% ± 10.9% (of 100%). A median of 7 months (range, 3-36 months) was observed to return to subjective maximum strength levels.

Before surgery, patients were able to bench a mean maximum of 162.03 ± 53.1 kg; at the time of the final follow-up, the maximum bench press had significantly decreased to a mean of 134.7 ± 52.1 kg (*P* = .001). Triceps press exercises reduced from a median of 70 kg (IQR, 50-85 kg) preoperatively to 60 kg (IQR, 35-70 kg) postoperatively (*P* = .001). An overall decrease of strength of 17.5% in triceps-demanding exercises was observed. In 14.3% of cases, patients mentioned a decrease of strength because of fear of retear. Ten patients (14.3%) reported cosmetic problems after surgery; the complaints were isolated to muscle contour.

### Complications and Failures

No intraoperative complications were noted. Revision surgery was performed in 7 cases; 6 cases (8.6%) for a rerupture and 1 case (1.4%) for postoperative infection with removal of material and repeated lavage.

## Discussion

The initial hypothesis was confirmed, as patients undergoing distal triceps tendon repair with a double-row reconstruction technique experienced beneficial clinical outcomes at midterm follow-up with a high return-to-sports rate and a high level of activity. Also, good cosmetic results with few subjective complaints were achieved after double-row reconstruction of the distal triceps.

In contrast to the high return-to-sports level, return to competition was limited, including regaining full force in triceps-demanding exercises. In this cohort, a relevant decrease of maximum force in triceps-demanding exercises was noted.

This finding may be explained by 2 main factors. First, as demonstrated in this study, a large proportion of patients reported preexisting triceps pain. This suggests that underlying tendon degeneration was already present before surgery, which could contribute to the postoperative loss of strength. Additionally, many patients expressed a fear of reinjury, which may have limited their performance during strength-demanding activities. Second, the reduced maximum force observed during triceps-heavy exercises may also be influenced by a high number of contralateral triceps tears. This possibility is particularly relevant given the high proportion of body building patients in this cohort—a group known to have a higher baseline risk of tendon injury compared with the general population.

Another point is the postoperative complaints about cosmesis. In this cohort, the main concern was about postoperative muscle belly deformities. Other literature names delayed wound consolidation as the main cosmetic concern after surgery.^6^ Given the highly athletic nature of our cohort, muscle contour is considered more important by the patient collective than the average population.

When referring to the surgical technique, the literature revealed clinical as well as biomechanical superiority of suture anchor reconstruction in terms of stability and retear rates in comparison with transosseous repair.^9,20,25,29^ In their meta-analysis, Tran et al^29^ demonstrated significantly lower general complications in suture anchor reconstruction for distal triceps reconstruction (8% vs 18% for transosseous repair; *P* = .008). They observed significantly lower retears after suture anchor reconstruction (7% transosseous repair vs 2% suture anchor reconstruction; *P* = .03).

The literature reveals favorable clinical results after suture anchor reconstruction for distal triceps tendon rupture, analogous to the current study.^1,7,15,16,31^ Waterman et al^31^ examined 69 distal triceps tendon ruptures with a minimum follow-up of 1 year. At the time of final follow-up, the patients had achieved a mean of 89.9 ± 14.6 points for the SANE score, 90.7 ± 29.7 points for the MEPS, and 9.7 ± 16.3 points for the QuickDASH score, with a mean VAS score of 0.9 ± 1.7.

Heikenfeld et al^15^ reported beneficial outcomes in terms of patient-reported outcome measures. They performed a prospective study including 14 patients after surgical treatment of the distal triceps tendon. At the final follow-up 12 months after surgery, the patients had achieved 96 points for the MEPS and 4.5 points for the QuickDASH score.

Further studies revealed promising data regarding return to sports.^2,12,19,24^ However, there were variable data about returning to full action, and precise specification of the sports participation level was not mentioned. Mair et al^19^ noted that 1 professional football player in their study returned to full play after 7 weeks, Finstein et al^12^ examined professional football players undergoing transosseous repair of the distal triceps tendon and observed a mean return to play of 165 days. Furthermore, they described no restrictions when returning to full stress. Agarwalla et al^1^ noted similar results to our study regarding return to sports. They were able to examine 81 patients with a minimum follow-up of 1 year after distal triceps reconstruction. Approximately 90% were able to return to at least one of the sports they participated in before surgery at a mean of 5.9 ± 4.4 months after surgery. Unlike our study, 29.5% of patients returned to a lower level of intensity.

Lastly, compared with the rerupture rates of 0% to 7%^16,29,31^ reported in the literature, we noted slightly more reruptures. However, our increased rerupture rate might be explained by the extended mean follow-up of 6 years in contrast to other studies with short-term follow-up and the highly selective and demanding patient collective. This cohort consisted of a highly athletic collective with great demands to their triceps muscle function, especially with a focus on the return to prior activity and return to competition in weight lifting. Moreover, returning to sports too early might have contributed to an increased failure rate.

### Limitations

This study has several limitations. First, the study inherits the associated biases of not personally examining the patients. We only sent out questionnaires when patients might have incorrectly answered some questions or when questions might have been over- or underestimated. Second, follow-up times varied between 31 and 141 months. Whereas those undergoing recent surgery were fully participating in sports, some patients undergoing surgery 10 years before had reduced their sports participation during the past years. Third, sports activity in terms of personal best form/peak force was also obtained through a questionnaire, meaning it is subjectively biased.

## Conclusion

Distal double-row reconstruction of triceps tendon ruptures achieved good clinical and cosmetic results with a low complication profile in this high-demand patient population consisting of (semi-)professional strength athletes. Subjectively, full strength was regained after a mean of 7 months; however, selective triceps strengths during the bench and triceps press resulted in significantly reduced weight loads postoperatively.
